# Long-term survival by repeat resection for metastases from primary retroperitoneal leiomyosarcoma: A case report

**DOI:** 10.1016/j.ijscr.2021.105891

**Published:** 2021-04-15

**Authors:** Nao Kitasaki, Tomoyuki Abe, Akihiko Oshita, Tsuyoshi Kobayashi, Shuji Yonehara, Hideki Ohdan, Toshio Noriyuki, Masahiro Nakahara

**Affiliations:** a Department of Surgery, Onomichi General Hospital, Onomichi, Hiroshima, Japan; b Department of Pathology, Onomichi General Hospital, Onomichi, Hiroshima, Japan; c Department of Gastroenterological and Transplant Surgery, Graduate School of Biomedical and Health Sciences, Hiroshima University, Hiroshima, Japan

**Keywords:** Primary retroperitoneal leiomyosarcoma, Long-term survival, Surgery

## Abstract

**Backgound:**

Retroperitoneal (RP) leiomyosarcoma (LMS) is a rare type of cancer, accounting for 0.1% of all malignancies. The gold-standard treatment for sarcoma is complete resection, and a 50% 5-year overall survival (OS) rate can be achieved by curative surgery. The survival benefits of radiotherapy and systemic chemotherapy for recurrence are not as good as those of surgical resection. To the best of our knowledge, there are a few reports that aggressive radical surgery significantly prolonged the survival period as our case. This case was reported in accordance with the SCARE 2020 Guideline (Ref).

**Case presentation:**

An 84-year-old woman was referred to our hospital for treatment of a primary RP tumour. At the age of 52-year-old, she underwent complete resection of an RP mass in 1991. Twenty-four years after the primary resection, metachronous recurrences occurred within the soft tissues, which were repeatedly resected. From 2015–2019, liver resections were performed thrice, and the patient survived with no signs of recurrence 1 year after the last surgery.

**Conclusion:**

Long-term survival of 29 years was achieved after undergoing over 20 surgical resections. Herein, we report the long-term survival of a patient who underwent repeated aggressive surgical resections for RP LMS recurrence anda literature review.

## Highlights

• Retroperitoneal (RP) leiomyosarcoma (LMS) is rare, with a high recurrence rate.
• A woman with RP LMS underwent over 20 surgeries for recurrence over 24 years.
• Long-term survival of 29 years was achieved after these resections.
• Aggressive and radical repeat resections may be beneficial in such patients.

## 1 Background

Retroperitoneal (RP) leiomyosarcoma (LMS) is a rare type of cancer that accounts for 0.1% of all malignancies [1]. RP sarcoma is reported in 10% of individuals with soft tissue sarcomas derived from the retroperitoneum. Liposarcoma, LMS, undifferentiated pleomorphic sarcoma, and solitary fibrous tumours have several unusual symptoms [2]. The gold standard treatment for sarcoma is complete resection, and a 50% 5-year overall survival (OS) rate can be achieved by curative surgery [3]; however, the recurrence rate is relatively high (>50%). The treatment for sarcoma recurrence remains unclear, and multiple metastases and unresectable states are associated with a poor prognosis [4,5]. If the recurrence site is completely resectable, the OS is improved after the curative resection [6]. The survival benefits of radiotherapy and systemic chemotherapy for recurrence are not as good as those of surgical resection. To the best of our knowledge, there are a few reports that aggressive radical surgery significantly prolonged the survival period, as in our case [7]. Herein, we report the long-term survival of a patient who underwent repeated aggressive surgical resections as RP LMS recurrence treatment and a literature review.

## 2 Case presentation

**Fig. 1:**
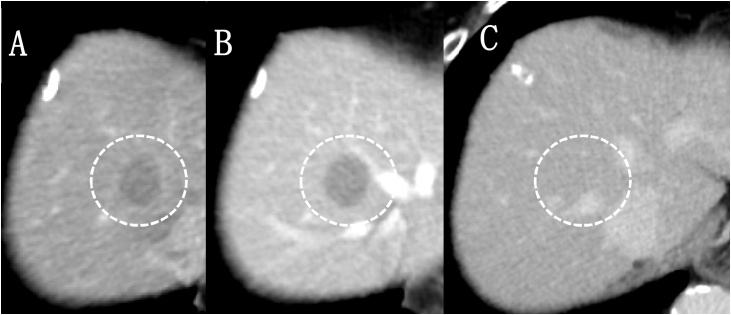
Dynamic abdominal computed tomography findings A: The arterial phase showing a ring-like enhanced mass at segment 7 measuring 35 mm in diameter, shaped like an irregular arrow. B: Portal phase. C: The enhancement is prolonged to the late phase.

**Fig. 2:**
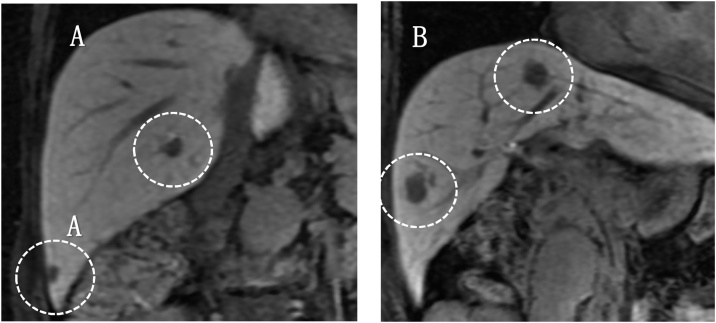
Findings of magnetic resonance imaging (MRI). A: The tumor shows a low signal intensity on a T1-weighted image. B: The tumor shows a high signal intensity on a T2-weighted image. C: At the early phase on ethoxybenzyl magnetic resonance imaging (EOB-MRI), the tumor shows ring-like enhancement. D: The enhancement is not detected throughout the tumor in the hepatocyte phase on EOB-MRI.

**Fig. 3:**
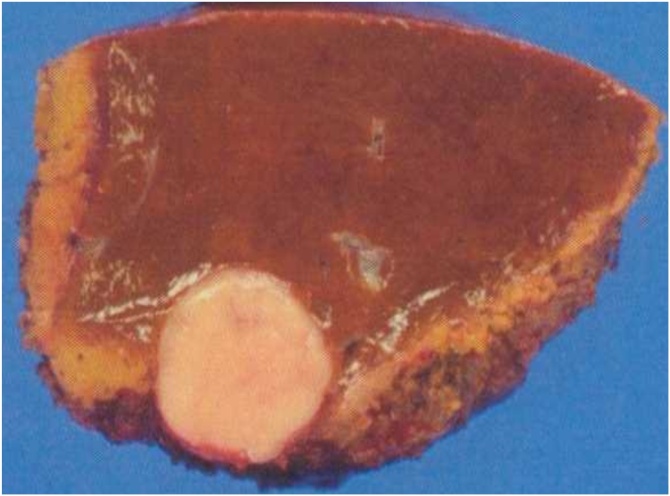
Imaging. A: Gross appearance of the cut surface showing a solid whitish mass measuring 35 mm × 32 mm × 30 mm with irregular margins. Clear cells are abundantly clear.

**Fig. 4:**
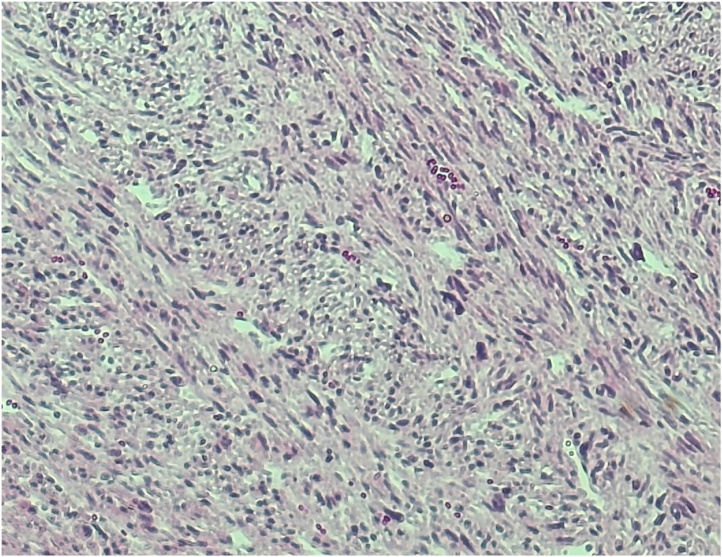
Histopathological findings: The tumor consists of poorly differentiated adenocarcinoma tissue and clear cells with atypical nuclei.

**Fig. 5:**
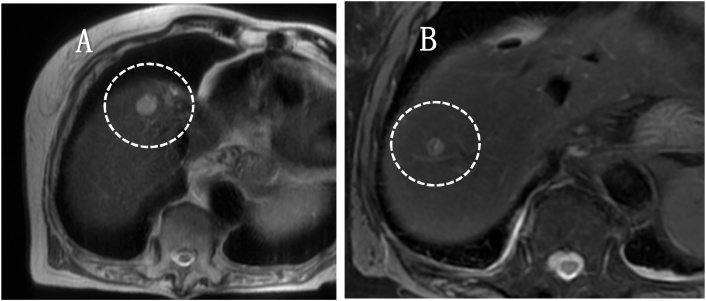
A: The tumour shows a high-signal intensity at segments 3 and 4 on a T2-weighted image in 2017. B: The tumour shows a high signal intensity at segment 8 on a T2-weighted image in 2019.

An 84-year-old woman was referred to our hospital for treatment of a primary RP tumour. At the age of 52, the patient underwent complete resection of a mass, approximately 3 cm at RP in 1991. The pathological diagnosis was LMS. Twenty-four years after the primary resection, metachronous recurrences occurred within the soft tissues, which were repeatedly resected. The recurrence sites were as follows: psoas muscle, latissimus dorsi muscle, deltoid muscle, and chest wall. As the tumours within the soft tissues felt stiff upon palpation, these tumours were easily detected by the patient. Ten recurrences occurred in the extremities, two in the retroperitoneum and three in the back. The chest wall was also involved twice, but both were completely resected. In 2015, multiple liver metastases were identified. Computed tomography(CT) showed that the tumour was located in segments 4, 5, 6, and 8. The whole tumour was enhanced in the early phase, and the enhancement was prolonged in the late phase (Fig. 1A–C). Ethoxybenzyl-magnetic resonance imaging(EOB-MRI) revealed that the tumours showed a high signal intensity on T2WI and a low signal intensity on T1WI (Fig. 2A and B). S4 subsegmentectomy and partial resection of sections 5,6, and 8, and cholecystectomy were performed. The operation time was 338 min, while intraoperative bleeding was measured in 400 ml. Gross examination revealed tumors with a fleshly and white-gray appearance and haemorrhagic foci (Fig. 3). Microscopically, the neoplastic cells appeared elongated with abundant cytoplasm and centrally located nuclei containing blunt-ended nucleoli resembling cigars (Fig. 4). Pathological examination revealed LMS metastasis. In 2017, liver metastasis recurred, and partial resection of segments 3 and 4 was performed (Fig. 5A). The patient was pathologically diagnosed with LMS metastases. In 2019, the patient underwent liver resection for the third time for segment 8 recurrence and survived with no signs of recurrence 1 year after the last surgery (Fig. 5B). Long-term survival of 29 years was achieved after undergoing over 20 surgical resections. All procedures used in this study were approved by the ethics committee of our institution. Written informed consent was obtained from the patient for the publication of this case report and accompanying images

## 3 Discussion

Metastasis after curative surgery for LMS occurs in approximately 40% of cases, and the most frequent site of metastasis is the lung via the hematogenous route. Our patient had more than 20 metachronous recurrences, and long-term survival was achieved by undergoing repeated aggressive surgeries. Except for three liver metastasis cases, all metastatic lesions were detected at the late stage, which we found on the extremities and back muscles. R0 resections were then repeatedly performed for metastasis. Previous studies have demonstrated that R1/2 resection is an independent risk factor for recurrence-free survival in patients with LMS [8,9]. Marudanayagam et al. reported that patients with liver metastasis from LMS are good candidates for liver resection among those with various types of soft tissue sarcomas [10]. Our patient experienced liver metastasis three times, which was detected at the late stage; the tumours did not invade the main Glisson and main hepatic veins. Konstationos et al. reported that repeat liver resections combined with multimodality treatment are essential in patients with metastatic LMS [11]. Our patient underwent repeated resections of recurrent metastases with three liver resections, and a survival benefit of 29 years was achieved.

The benefits of surgical resection for extrahepatic metastasis remain unclear; the incidence of local recurrence was reported to be approximately 50%, while that of distant metastasis was approximately 40% after curative surgery [12]. Lang et al. showed that liver resection timing had no influence on survival in patients with extrahepatic tumours if complete removal of both liver metastases and extrahepatic tumour is achieved [8]. In another study, repeated resections for recurrent cases have been reported to reduce the local recurrence rate; however, this was not associated with improved OS [13]. Grobmyer et al. reported that repeated complete resections in patients with local recurrences could provide the same therapeutic effect as experienced by the group that survived without recurrence after undergoing complete resection. Resection should be considered in patients with first and subsequent local recurrences (even if multifocal) of RP LMS because it is associated with improved survival [14]. Resection is recommended in patients with local recurrence, but the benefit of partial resection in patients with distant metastases remains uncertain. The number of recurrences and the frequency of complete resection of locoregional recurrence can influence the survival rate. However, locoregional recurrence is less frequent than distant metastasis; hence, complete resection of recurrent lesions can only be performed in limited conditions [15].

Until now, the efficacy of systemic chemotherapy for the recurrence of LMS remains unclear. Chemotherapy consisting of cyclophosphamide, doxorubicin, and dacarbazine is considered for patients with unresectable or distant metastases, but these patients' prognosis remains poor. Recent retrospective studies have demonstrated that chemotherapy administration in combination with surgery preoperatively or postoperatively may result in a worse survival outcome compared with surgery alone. Some cases have shown that systemic chemotherapy prolongs long-term survival [16]. In some reports, salvage surgery for recurrent RP LMS after chemotherapy was associated with improved OS [12,17,18]. In this case, postoperative chemotherapy or radiation therapy was not provided; instead, surgery was performed. Approximately 20 resections could be performed because the patient had resectable lesions. Chemotherapy and radiotherapy have also been reported to control lesions [16], and long-term survival is expected when this treatment is combined with surgical resection.

Deciding whether to resect for recurrent cases and the timing of resection with the possibility of concomitant non-surgical treatment (such as radiation or systemic therapy) can be difficult. Surgery in patients with recurrent RPS can be more technically challenging than surgery in patients with primary disease. Adhesions in the intestine and other parts of the body often need to be resolved, which increases the risk of complications, such as intussusception and postoperative ileus. In general, each recurrence of RPS is associated with a lower complete resection rate and worse clinical outcomes. Therefore, it is important to appropriately select patients with recurrent RPS expected to benefit the most from re-resection. Important considerations favouring re-resection include R0 resection, low-grade tumours, a disease-free interval, and unifocal rather than multifocal local disease [19].

Even in cases where R0 resection is not possible, resection for symptomatic relief may be considered, but take into account the fact that systemic chemotherapy may be interrupted. The duration of symptomatic relief has been reported to be short [20]

The decision-making process for palliative RPS resection is complex, and cases should be discussed among multidisciplinary team members.

In conclusion, repeat R0 surgery for RP metastases can provide long-term survival of 30 years after the primary surgery. Regardless of the lack of an established treatment strategy for recurrent RP metastases, aggressive surgical resection should be considered in patients with resectable RP metastasis.

## Declaration of Competing Interest

The authors report no declarations of interest.

## Sources of funding

No.

## Ethical approval

This paper has been granted ethical approval.

## Consent

Written informed consent was obtained from the patient for publication of this case report and accompanying images. A copy of the written consent is available for review by the Editor-in-Chief of this journal on request.

## Author’s contribution

Nao Kitasaki is the first author, and Tomoyuki Abe is a corresponding author.

Nao Kitasaki, Tomoyuki Abe and Akihiko Oshita contribute the study concept and design.

Nao Kitasaki, Tomoyuki Abe, Shuji Yonehara, Toshio Noriyuki and Masahiro Nakahara contribute the data collection, data analysis and interpretation.

Nao Kitasaki, Tomoyuki Abe, Tsuyoshi Kobayashi and Hideki Ohdan contribute the witing the paper.

## Registration of research studies

Not applicable.

## Guarantor

Tomoyuki Abe.

## Provenance and peer review

Not commissioned, externally peer-reviewed.
